# Identification of key biomarkers in breast cancer based on bioinformatics analysis and experimental verification

**DOI:** 10.1186/s43046-025-00260-8

**Published:** 2025-02-24

**Authors:** Yu Huan, Ping She, Xushan Cai, Jiehua Qi, Chunli Zhang

**Affiliations:** 1 Department of Clinical Laboratory, Jiading District Maternal and Children Health Hospital, Shanghai, 201821 People’s Republic of China; 2 School of Computer Science and Technology, Xi’an Jiaotong University, Xi’an, Shanxi 710049 People’s Republic of China

**Keywords:** DEGs, GO and KEGG, PPI, Experimental verification, IHC

## Abstract

**Background:**

Breast cancer (BC) is a malignant tumor characterized by a high incidence rate and is the leading cause of cancer-related deaths among women worldwide. This study aims to identify key genes and potential prognostic biomarkers using a bioinformatics approach.

**Methods:**

Three microarray datasets, GSE86374, GSE120129, and GSE29044, were downloaded from the GEO database. GEO2R and Venn diagram software were employed to identify differentially expressed genes (DEGs), while DAVID was utilized for functional enrichment analysis. Subsequently, STRING and Cytoscape were used to construct the protein–protein interaction (PPI) network among the DEGs. UALCAN, GEPIA, and the Kaplan–Meier plotter were employed for prognostic analysis. Following this, the correlations and alterations of key genes were examined using cBioPortal. Finally, immunohistochemistry (IHC) was performed to validate the expression levels of the key genes.

**Results:**

A total of 323 differentially expressed genes (DEGs) were identified. From the protein–protein interaction (PPI) network, 37 hub genes were selected. Validation using UALCAN, GEPIA, and Kaplan–Meier plotters revealed that three key genes—*RACGAP1*, *SPAG5*, and *KIF20A*—were significantly overexpressed and associated with poor prognosis in breast cancer (BC), as well as advanced tumor staging. The correlations and alterations of these key genes, as demonstrated on cBioPortal, indicated that their alterations co-occurred. Experimental verification through immunohistochemistry (IHC) confirmed that the proteins of these key genes were highly expressed in tumor tissues.

**Conclusions:**

The key genes identified in this study can enhance our understanding of the molecular mechanisms underlying breast cancer (BC). Additionally, these genes may serve as potential sensitive biomarkers for patients with BC.

## Introduction

Based on statistics published by the International Agency for Research on Cancer (IARC), breast cancer (BC) has the highest incidence rate of any cancer worldwide as of 2020 [1]. In 2021, BC accounted for 30% of all new diagnoses of female cancers [2], and it is projected to be the most common cancer among females in both China and the USA [3]. A growing body of evidence suggests that the carcinogenesis and progression of BC are linked to the abnormal expression and mutation of specific genes, including breast cancer gene 1 (BRCA1), breast cancer gene 2 (BRCA2), B-cell lymphoma 2 (Bcl-2), protein 53 (p53), epidermal growth factor receptor (EGFR), and various cytokeratins (such as CK5/6, CK14, CK17) [4–6]. Despite advancements in modern diagnostic and therapeutic methods, the mortality rate of BC remains high due to recurrence, metastasis, and drug resistance. Early detection of BC in patients is crucial for effective treatment and for the longitudinal analysis of cancer evolution in response to therapy. Therefore, identifying alterations in new biomarkers associated with BC could provide valuable insights into the underlying mechanisms of the disease and facilitate the development of novel therapeutics.

In light of advancements in microarray technology and bioinformatics methods, we are gaining deeper insights into the molecular mechanisms underlying tumor development [7–9]. In the present study, we first downloaded three profiles—GSE86374, GSE120129, and GSE29044—from the Gene Expression Omnibus (GEO). We then utilized GEO2R and DAVID to identify differentially expressed genes (DEGs) between breast cancer (BC) tissues and normal tissues, resulting in the identification of 184 up-regulated and 139 down-regulated DEGs. Secondly, Gene Ontology (GO) and Kyoto Encyclopedia of Genes and Genomes (KEGG) pathway enrichment analysis were executed to comprehend the molecular mechanisms prospective occurrence and development of BC. Thirdly, 37 hub genes which may be candidate biomarkers for BC were discovered by protein–protein interaction (PPI) network technique, Subsequently, only three key genes were elected by UALCAN, GEPIA, and Kaplan–Meier plotter. Finally, the cBioPortal online platform was involved in the analysis of expressions and mutations in BC, and immunohistochemistry (IHC) was used to verify the expressions of the key genes. To sum up, DEGs and hub genes distinguished in this study can help us better understand the molecular mechanisms fundamental to the carcinogenesis and progression of BC and provide some latent sensitive biomarkers for BC patients.

## Materials and methods

### Data source and processing

GEO (http://www.ncbi.nlm.nih.gov/geo/) [10] is a free gene expression database that stores massive various high-throughput experimental data. Three gene expression datasets were downloaded from GEO, namely, GSE86374, GSE120129, and GSE29044 [11, 12]. GSE86374 contained 123 BC tissues and 36 normal breast tissues, GSE120129 contained 30 BC tissues and 53 normal breast tissues, and GSE29044 contained 73 breast cancer (BC) tissues and 36 normal breast tissues. GSE86374 bottomed on the GPL6244 platform, and the other two both bottomed on the GPL570 platform.

### Classification of DEGs

GEO2R (http://www.ncbi.nlm.nih.gov/geo/geo2r) is a software that can emerge with differential analysis of expression profile chips based on the GEO database. DEGs between tumor and normal tissue in the breast of the three databases above were made out by GEO2R. The criteria for identifying DEGs were based on LogFC (fold change) ≥ 1 or *LogFC* ≤ − 1, and *P*-value < 0.05 were deemed to be statistically significant. Whereupon, the general DEGs in the three separate cohorts were picked out by Venn (http://bioinformatics.psb.ugent.be/webtools/Venn/).

### GO and KEGG analyses

DAVID (https://david.ncifcrf.gov/) is not only a biological database but also an online analysis software, which can be used for distinguishing gene function differences and pathway enrichment [13, 14]. GO analysis in DAVID can be divided into different analysis modules of gene functions [15], for instance, biological process (BP) includes processes completed through multiple molecular activities occurring in biomolecules, cells, tissues, and so on, molecular function (MF) includes the activities of a single gene product such as protein and RNA or a complex of multiple genes products at the molecular level, and cellular component (CC) means the cellular structural location of a gene when it is executing its function. KEGG analysis in DAVID is often applied to clear the related functions and action pathways of differentially expressed genes [16]. In this study, DEGs enrichment of BP, CC, MF, and the KEGG pathways was analyzed. *P* < 0.01 (*P*-value) was considered statistically significant.

### PPI network construction and hub gene selection

The PPI network of DEGs was built by the STRING (version 11.5) database (https://string-db.org) [17], and the screening criteria were the combined score ≥ 0.9. Then, the data filtered above was loaded to Cytoscape (version 3.7.2), which is a free bioinformatics software for graphically displaying molecular interaction [18, 19] to draw a map of DEGs’ networks. MCODE is an application in Cytoscape that clusters a given network based on topology to discover densely connected areas. The most significant module, in other words, hub genes in the PPI networks, was picked out by MCODE. MCODE scores > 5, degree cutoff = 2, node score cutoff = 0.2, max depth = 100, and k-score = 2 as selection criteria.

### Key genes election and analysis

Both UALCAN (http://ualcan.path.uab.edu/cgi-bin/ualcan-res.pl) and GEPIA (http://gepia.cancer-pku.cn/) are comprehensive, user-friendly, and interactive web resources for analyzing cancer omics data [20, 21]. Open cancer omics data such as TCGA and MET500 can be easily accessed using the two databases [22]. In the study, UALCAN was devoted to deciding whether the hub genes with poor survival and GEPIA were used to analyze, whether these genes have different expressions between tumor and normal tissues, and whether these were based on different subclasses of BC. The Kaplan–Meier plotter can evaluate the impact of more than 54,000 genes in 21 types of tumors [23] (https://kmplot.com/analysis/), and it is the most authoritative survival analysis database. In this study, a Kaplan–Meier plotter was used to discover OS and RFS among BC patients of the key genes.

### The correlations and alterations of key genes on cBioPortal

The cBioPortal (https://www.cbioportal.org/) website which contains large-scale tumor research projects such as TCGA and ICGC integrates the data of more than 100 tumor genome studies [24]. cBioPortal has powerful analysis functions, including OncoPrint-gene mutation map, plots analysis of the relationship between copy number variation, and gene mutation or gene expression, mutations—gene mutation list, network—coexpression network, and other analysis results. In this study, co-expression and mutations of the key genes were analyzed by cBioPortal.

### The experimental verification of key genes with immunohistochemistry (IHC)

IHC was performed to assess the expressions of the key genes. Tumor and paired paracancerous tissues of 20 BC patients were gathered from patients who received treatment at Jiading District Maternal and Children Health Hospital, Shanghai, from March to June 2023. The antibodies of key genes were from Proteintech Group, Inc. Wuhan, China. The experiment was conducted on a fully automated IHC machine (LEICA, BOND-MAX), and the dilution ratios of antibodies of RACGAP1, SPAG5, and KIF20A were respectively 1:150, 1:300, and 1:300.

### Inclusion criteria

All patients with breast cancer are diagnosed as breast cancer for the first time, and have not received any chemotherapy, radiotherapy, or biological treatment.

### Exclusion criteria

These are patients with concurrent malignant tumors and incomplete clinical and pathological data.

## Results

### Classification of DEGs BC

**Fig. 1:**
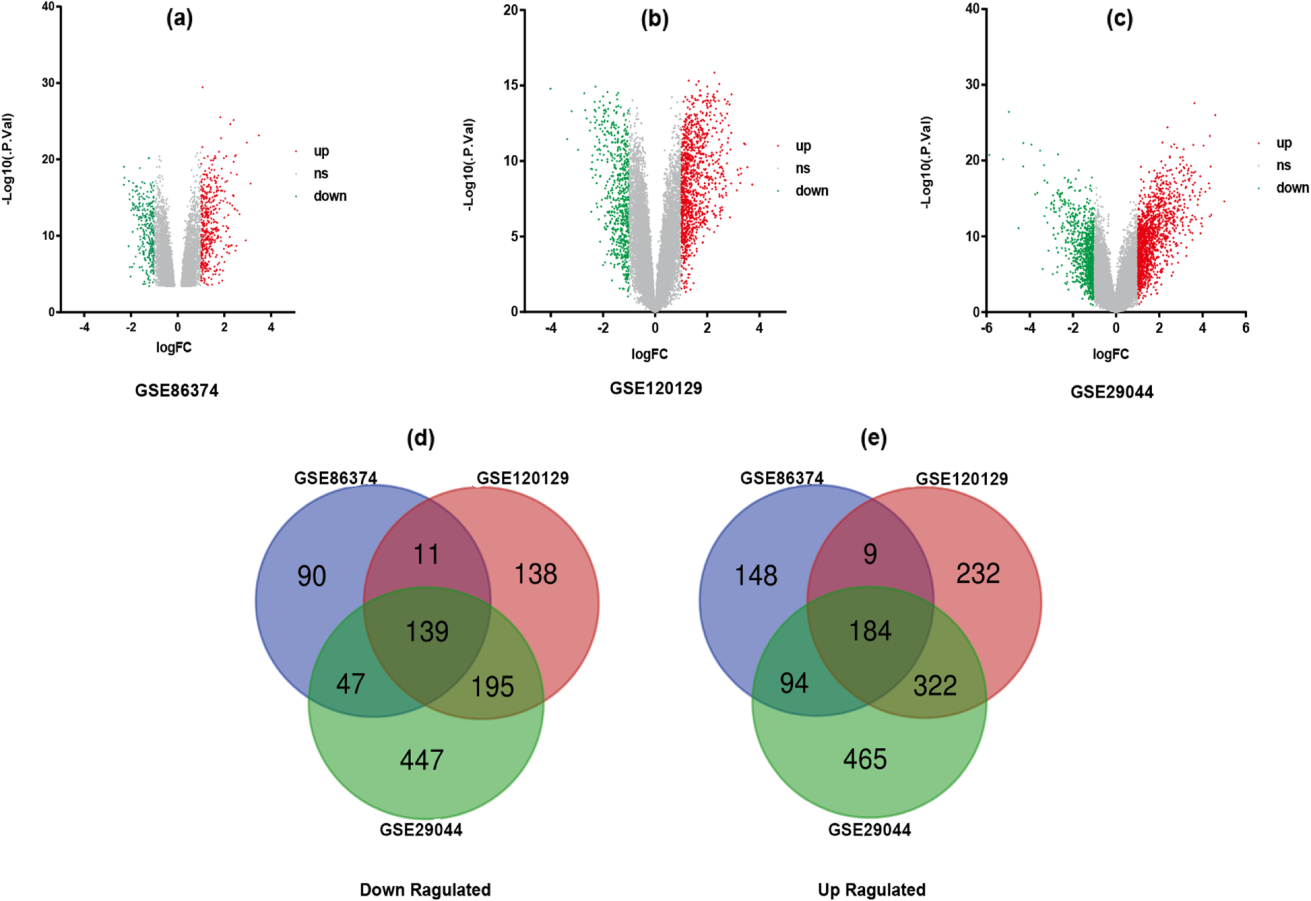
DEGs of three GEO profiles. **a**, **b**, **c** The volcano plot of DEGs of the three profiles. **d**, **e** Down-regulated and up-regulated genes of the Venn diagram of the three GEO profiles

After analyzing respectively, three gene expression profiles (GSE86374, GSE120129, and GSE29044) from GEO were downloaded. A total of 449, 1078, and 1427 up-regulated and 300, 639, and 1037 down-regulated genes were screened out respectively from GSE86374, GSE120129, and GSE29044. The volcano plot of DEGs of each profile is shown in Fig. 1a, b, and c. And 184 up-regulated and 139 down-regulated DEGs were picked out by Venn software (Fig. 1d, e).

### GO and KEGG analyses

**Fig. 2:**
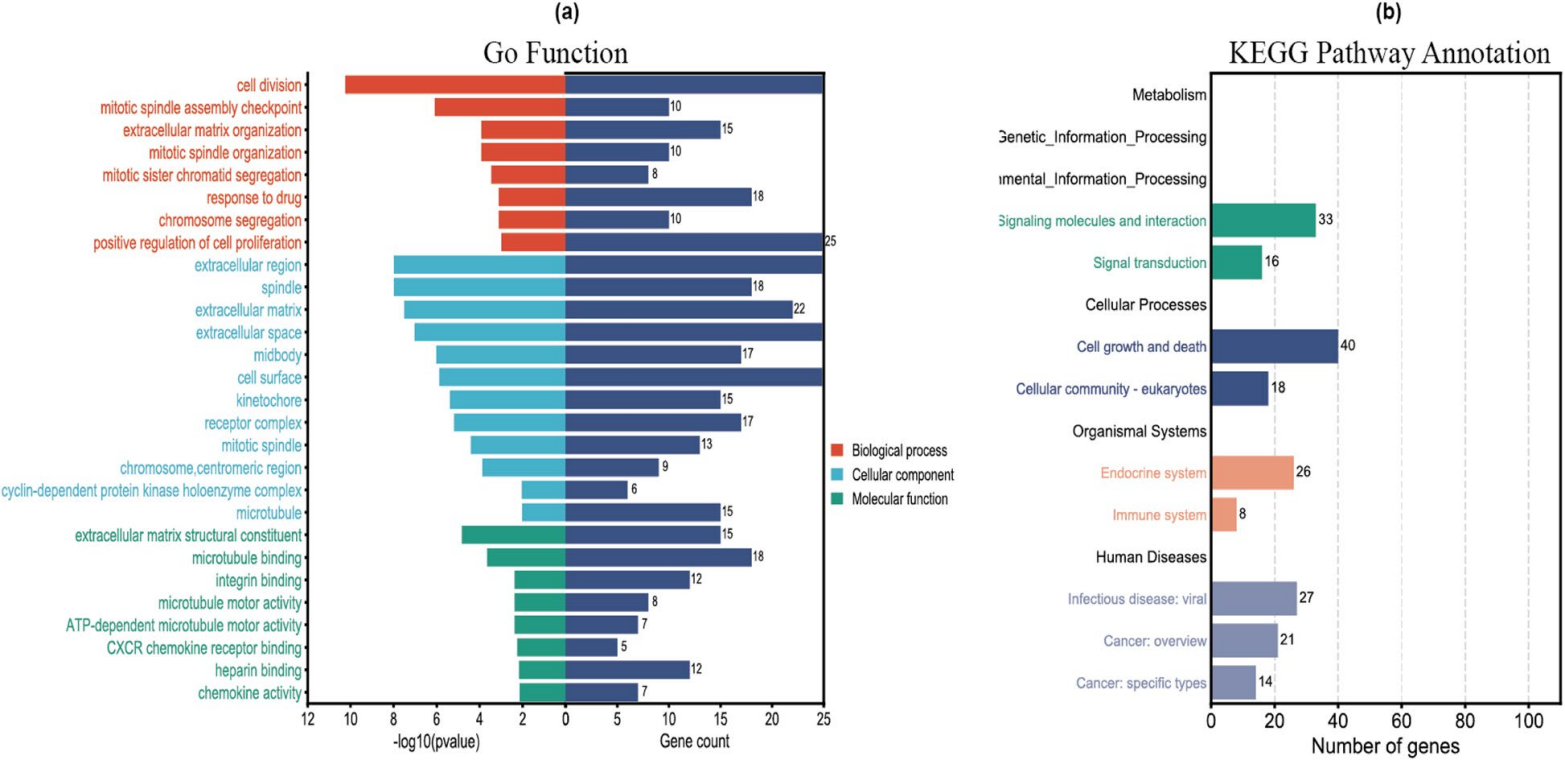
GO and KEGG pathway analysis. **a** GO function analysis of the DEGs. **b** KEGG pathway annotation of the DEGs

All DEGs enrichment score was analyzed by online DAVID software. GO analysis results indicated that modifications in biological processes (BP) of DEGs were remarkably beneficiated in cell division, mitotic spindle assembly checkpoint, extracellular matrix organization, mitotic spindle organization, mitotic sister chromatid segregation, response to drug, chromosome segregation, and positive regulation of cell proliferation. Modifications in cell component (CC) of DEGs were mainly beneficiated in the extracellular region, spindle, extracellular matrix, extracellular space, midbody, cell surface, kinetochore, receptor complex, mitotic spindle, chromosome centromeric region, cyclin-dependent protein kinase holoenzyme complex, and microtubule. Modifications in molecular function (MF) were mainly beneficiated in extracellular matrix structural constituent, microtubule binding, integrin binding, microtubule motor activity, ATP-dependent microtubule motor activity, CXCR chemokine receptor binding, heparin binding, and chemokine activity. And KEGG pathway analysis manifested that the DEGs were mainly focused on signaling molecules and interaction, signal transduction, cell growth and death, cellular community — eukaryotes, immune system, infectious disease—viral, endocrine system, cancer-specific types, and cancer—overview (Fig. 2).

### PPI network construction and hub gene selection

**Fig. 3:**
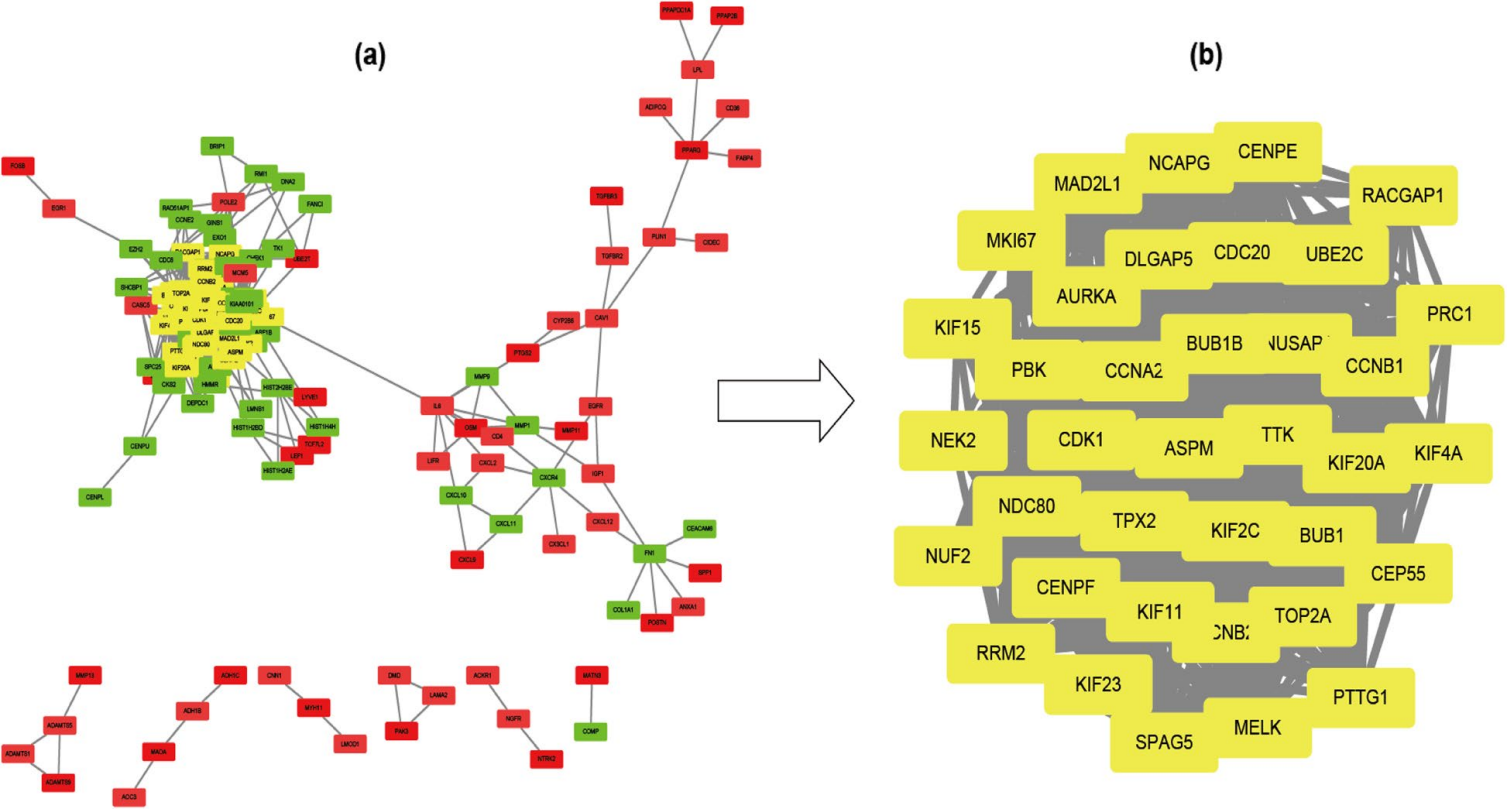
DEGs PPI network built by STRING and Cytoscape. **a** Three-hundred twenty-one nodes and 1031 edges in the PPI network. Red circles meant up-regulated genes, green circles meant down-regulated ones, and yellow circles meant hub genes. **b** Hub genes of DEGs picked out by MCODE in Cytotype

All of the DEGs were imported into STRING software to acquire information on interactivities between every two proteins. There were 321 nodes, and 1031 edges were obtained after analysis in the PPI network. Afterward, all the data of nodes and edges were downloaded and imported to Cytotype. After setting degree cutoff = 2, node score cutoff = 0.2, K-core = 2, and max depth = 100 as selection criteria, 37 hub genes in these nodes were selected with the help of MCODE which is one of the Cytotype application (Fig. 3, Table 1).

**Table 1:** The survival probability information of the 37 hub genes

Category	Official gene symbol					
Genes with significantly worse survival (*P* < 0.01)	*RACGAP1*		*SPAG5*		*KIF20A*	
Genes with significantly worse survival (*P* > 0.05)	*MAD2L1*	*NCAPG*	*CENPE*	*MKI67*	*AURKA*	*RRM2*
	*DLGAP5*	*CDC20*	*UBE2C*	*KIF15*	*PBK*	*KIF23*
	*CCNA2*	*BUB1B*	*NUSAP1*	*CCNB1*	*PRC1*	*MELK*
	*NEK2*	*CDK1*	*ASPM*	*TTK*	*KIF4A*	*PTTG1*
	*NDC80*	*TPX2*	*KIF2C*	*BUB1*	*CEP55*	
	*NUF2*	*CENPF*	*KIF11*	*CCNB2*	*TOP2A*	

### Key genes election and analysis

**Fig. 4:**
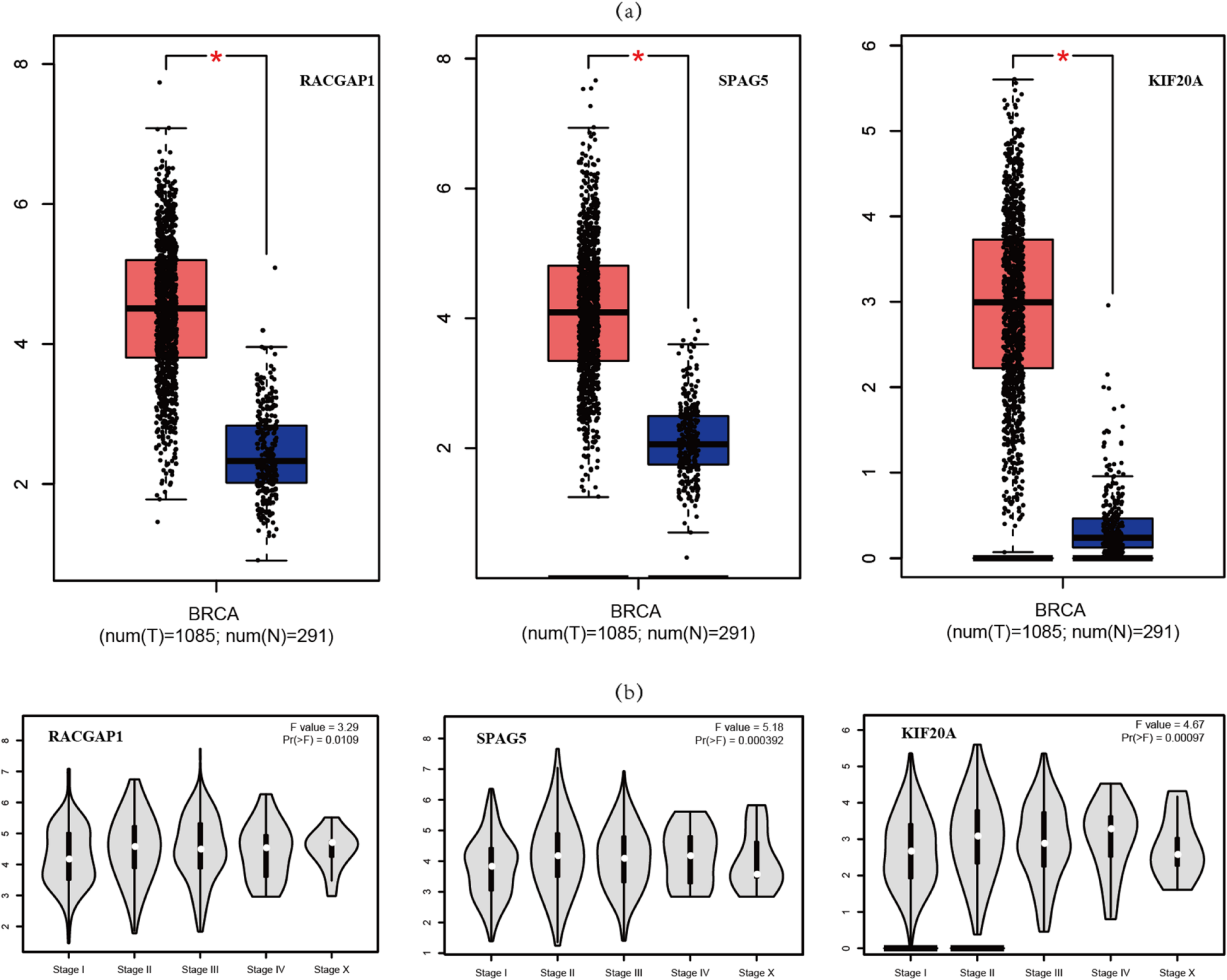
The expression of *RACGAP1*, *SPAG5*, and *KIF20A* in BC. **a** The expression levels of *RACGAP1*, *SPAG5*, and *KIF20A* in BC tissues and breast normal tissues (*P < 0.05)). **b** Relevance between gene expression and different tumor stages in BC patients

All the 37 hub genes were unpacked by UALCAN about their survival probability, and the results showed that 3 of them had a significantly worse probability in BC patients (Table 1, *P* < 0.05). They were RACGAP1, SPAG5, and KIF20A. Then, the results of their expression level between BC and breast normal tissues by GEPIA indicated that the expression levels of RACGAP1, SPAG5, and KIF20A were all higher in BC tissues than in breast normal tissues, and the results had significant differences (Fig. 4a, P < 0.05). The expression of these three genes with different tumor stages of BC was also analyzed by GEPIA; all of them significantly varied too (Fig. 4b, P < 0.05).

**Fig. 5:**
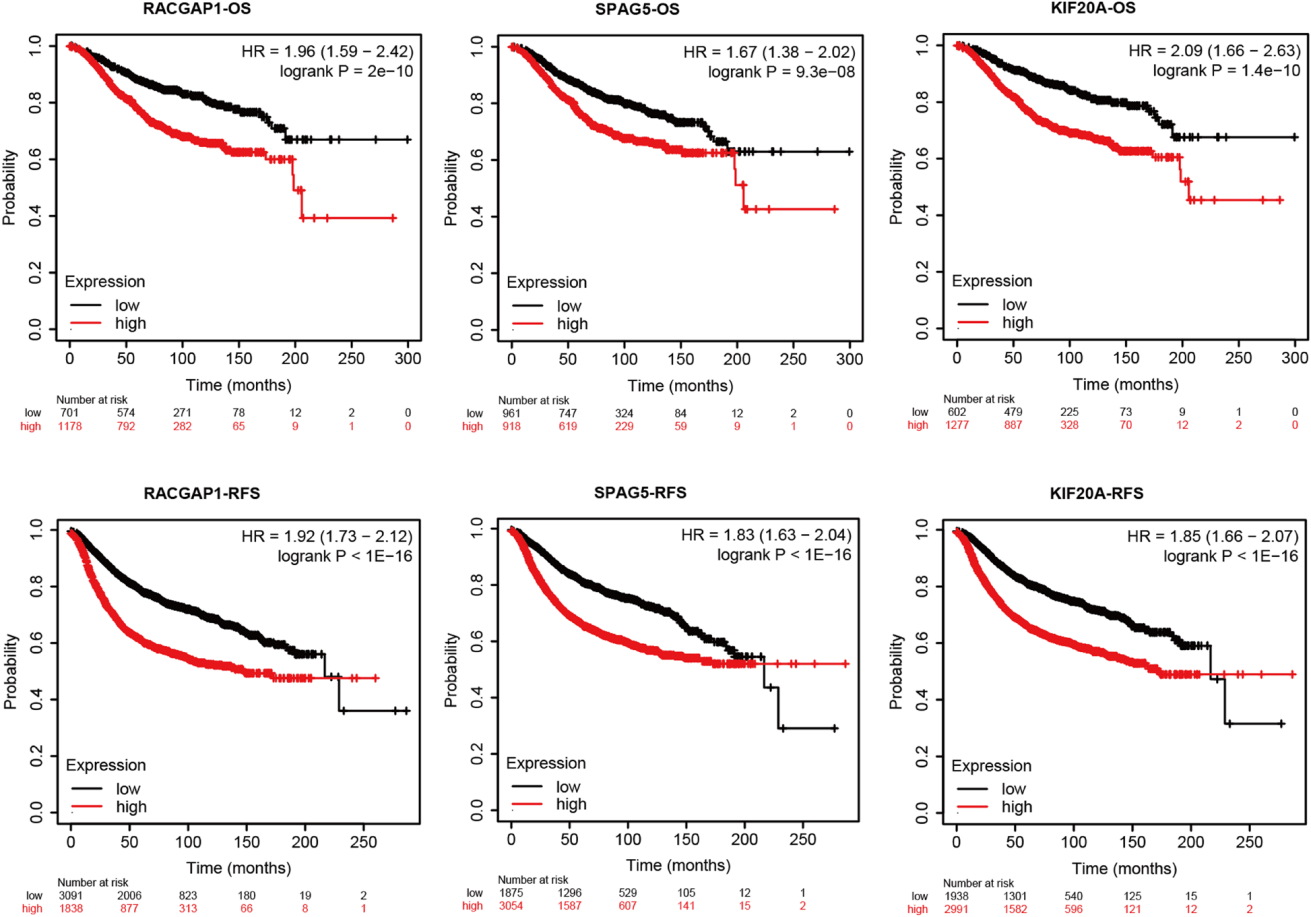
Prognostic information of *RACGAP1*, *SPAG5*, and *KIF20A* in BC. KM plotter of *RACGAP1*, *SPAG5*, and KIF20A in BC, and all of them had significantly worse survival. OS, overall survival; RFS, relapse-free survival (*P* < 0.05)

The Kaplan–Meier curve and log-rank test analyses disclosed that the increased RACGAP1, SPAG5, and KIF20A mRNA levels were closely related to the overall survival (OS) and relapse-free survival (RFS) and would predict worse prognosis of BC patients (*P* < 0.05) (Fig. 5).

### The correlations and alterations of key genes on cBioPortal

**Fig. 6:**
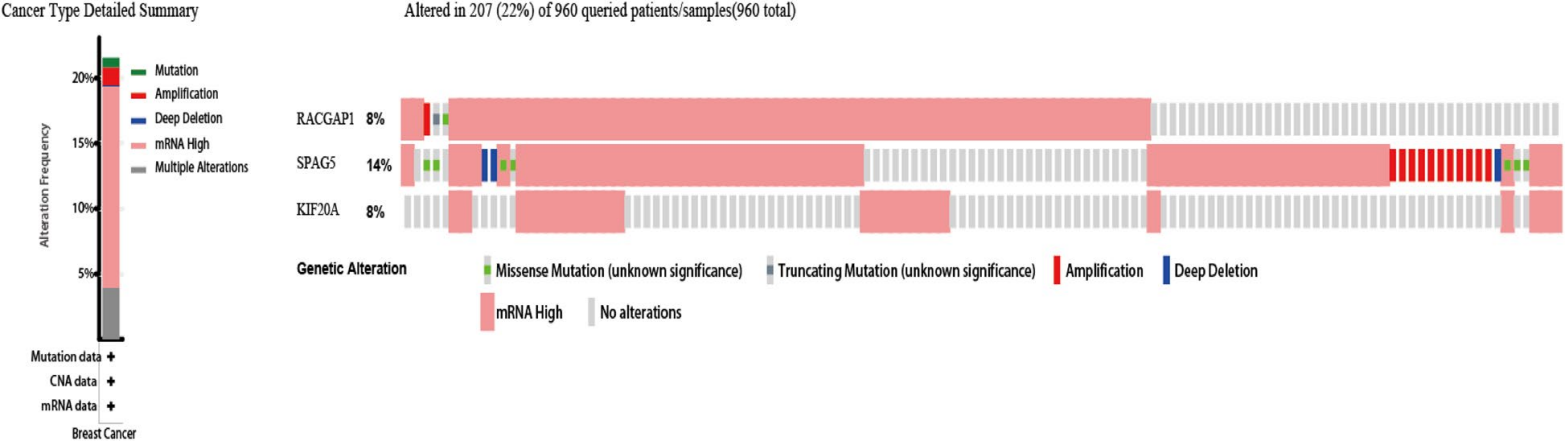
Gene expression and mutation analysis in BC. Alterations (mutation, amplification, deep deletion, mRNA high, multiple alterations) of *RACGAP1*, *SPAG5*, and *KIF20A* were detected in almost 22% of the samples (207 samples)

The correlations and alterations of *RACGAP1*, *SPAG5*, and *KIF20A* were analyzed by cBioPortal in breast invasive carcinoma (TCGA, Firehose Legacy). The 3 genes were changed in 960 samples of 1108 patients of BC (86.6%). Two or more alterations were discerned in approximately 22% of the samples (Fig. 6). Another analysis tested three pairs between the three tracks in the OncoPrint which is one of the analysis functions in cBioPortal indicating that the alterations co-occurred with each other (Table 2, *P* < 0.05).

**Table 2:** The assay tested three pairs between the three tracks in the OncoPrint

A	B	Neither	A not B	B not A	Both	Log2 odds ratio	*p*-value	*q*-value	Tendency
RACGAP1	SPAGS	790	32	92	46	> 3	< 0.001	< 0.001	Co-occurrence
RACGAP1	KIP20A	830	56	52	22	2.649	< 0.001	< 0.001	Co-occurrence
SPAG5	KIP20A	776	110	46	28	2.102	< 0.001	< 0.001	Co-occurrence

### The experimental verification of key genes with IHC

**Fig. 7:**
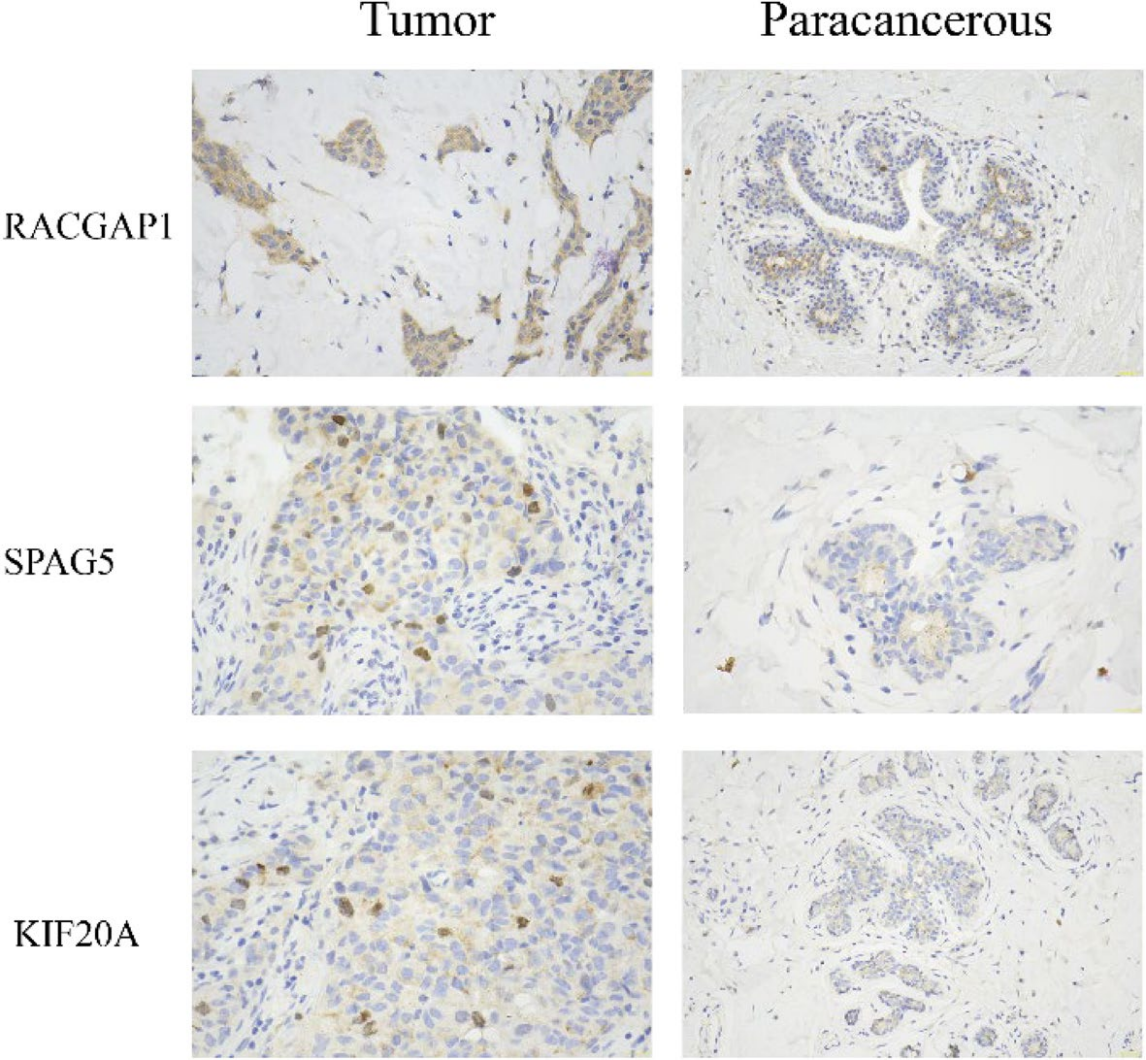
The experimental verification of RACGAP1, SPAG5, and KIF20A with IHC. RACGAP1, SPAG5, and KIF20A proteins were all highly expressed in tumor tissues compared with paired paracancerous tissues (× 40)

To prove the results of bioinformatics analysis, tumor and paired paracancerous tissues of 20 BC patients were used to simply verify with IHC. The results showed that compared with the adjacent tissue, RACGAP1, SPAG5, and KIF20A proteins were highly expressed in tumor tissues. RACGAP1 was expressed in the cytoplasm, while SPAG5 and KIF20A were expressed in the nucleus (Fig. 7).

## Discussion

Breast cancer has emerged as the leading cause of high incidence and mortality among women, surpassing lung cancer in recent years [25]. However, the pathogenesis of breast cancer remains unclear and may be influenced by factors such as the patient’s age, lifestyle habits, family history, genetic mutations, and hormonal levels. Therefore, investigating the molecular mechanisms underlying the physiology and pathology of breast cancer is essential for a comprehensive understanding of the disease’s occurrence, progression, and prognosis, as well as for informing clinical judgment and treatment strategies. High-throughput microarray and bioinformatics approaches have enabled researchers to explore genetic alterations in breast cancer, proving to be effective methods for identifying new biomarkers across various diseases. In this study, our short-term goal is to conduct follow-up experiments based on the results of bioinformatics analysis and preliminary experiments, while our long-term objective is to identify molecular markers that can aid in the prediction and treatment of breast cancer.

It is possible for the analysis of a single microarray dataset to overfit the data, resulting in low training error but high test error. For this reason, this study conducted a comprehensive analysis of 3 microarray datasets (GSE86374, GSE120129, and GSE29044), which included 226 breast cancer (BC) tissue samples and 175 normal breast tissue samples. Consequently, 323 differentially expressed genes (DEGs) were identified across the three microarray datasets, comprising 139 down-regulated genes and 184 up-regulated genes. Gene Ontology (GO) analyses indicated that the alterations in the gene modules primarily focused on cell division, extracellular regions, spindles, extracellular matrices, and extracellular matrix structural constituents. Kyoto Encyclopedia of Genes and Genomes (KEGG) enrichment analysis of the DEGs revealed that they were mainly associated with signaling molecules and interactions, as well as cell growth and apoptosis. Additionally, 37 hub genes were identified as the most significant components within the protein–protein interaction (PPI) network. Our findings suggest that these enhanced modules and pathways have a genetic impact on breast cancer. Furthermore, the predictive genes within these datasets may be interconnected and jointly regulate breast cancer within the network.

We identified three key genes associated with very low survival rates in breast cancer (BC) across these three datasets: *RACGAP1*, *SPAG5*, and *KIF20A*. *RACGAP1* (Rac GTPase-activating protein 1) is a component of the central spindle protein complex and encodes a GTPase-activating protein. This protein interacts with the active form of Rho GTPases and promotes GTP hydrolysis, which can lead to the negative regulation of Rho-mediated signal transduction. Numerous studies have indicated that *RACGAP1* is a highly expressed gene linked to poor prognosis in several types of human cancer [26–28]. Bioinformatics analyses have demonstrated that *RACGAP1* is significantly overexpressed in BC tissues associated with poor prognosis [29]. Additionally, analyses of online databases revealed a substantial increase in RACGAP1 mRNA expression across various tumor tissues [30, 31], which aligns with our findings.

*SPAG5* (sperm-associated antigen 5) encodes a protein that may play a crucial role in the function and dynamic regulation of mitotic spindles. The *SPAG5* gene is overexpressed in various types of tumor tissues, and elevated expression levels have been associated with poorer prognoses, as indicated by bioinformatics analyses. It has been observed that *SPAG5* exhibits carcinogenic activity as a spindle-related protein during mitosis in both solid tumors and hematological malignancies [32–34]. In triple-negative breast cancer (TNBC), the expression levels of *RAD51*, *BRCA1*, and *BRCA2* have been positively correlated with SPAG5 expression [35].

*KIF20A*, a member of the kinesin family of motor proteins, is localized in several cellular components, including the cleavage furrow, intercellular bridge, and midbody. It exhibits protein kinase binding activity and plays a crucial role in the formation of microtubule bundles, midbody abscission, and the regulation of cytokinesis. The expression of *KIF20A* has been associated with overall survival rates in various human cancers, including kidney cancer, prostate cancer, fibrosarcoma, and hepatocellular carcinoma [36–39]. Additionally, *KIF20A* can bind to microRNAs, which may influence the efficacy of chemotherapy in breast cancer cells [40].

Through a series of bioinformatics methods, we identified three genes: *RACGAP1*, *SPAG5*, and *KIF20A*. We conducted a preliminary validation of these genes using immunohistochemistry (IHC), and the results corroborated our bioinformatics analysis. Further literature searches revealed that the interactions between breast cancer and these key genes have not been extensively documented.

Despite the valuable insights provided by this study, it has several limitations. Firstly, the experiments conducted utilized immunohistochemistry, which revealed weak labeling of the three selected biomarkers. To achieve more effective detection of protein expression, we recommend employing methods such as Western blotting (WB) or immunofluorescence assays. While immunofluorescence is generally more effective due to the incorporation of fluorescent dye labeling, which produces stronger signals than immunohistochemistry, the research team in this study was not qualified to utilize this method. Secondly, the mechanisms by which these biomarkers influence breast cancer (BC) were not clarified, indicating that the relationships of these genes with BRCA1, BRCA2, and other BC-related genes warrant further investigation. Thirdly, although bioinformatics analysis is a powerful tool for understanding molecular mechanisms and identifying potential biomarkers, additional experimental validation is needed at the molecular, cellular, and biological levels. Fourthly, the roles these genes play in diagnosis were not adequately explored. Consequently, further investigations should focus on elucidating the underlying mechanisms connecting BC and these key genes.

## Conclusions

The current study aimed to identify differentially expressed genes (DEGs) in breast cancer (BC) that may be involved in its onset and progression. We identified a total of 323 DEGs, of which 184 were upregulated and 139 were downregulated. Among these, we identified 37 hub genes, including 3 key genes that could serve as diagnostic biomarkers for BC. The findings of this study provide new insights into potential strategies for the diagnosis, prognosis, and targeted treatment of breast tumors. However, we acknowledge that a primary limitation of this study is the reliance on a single experimental verification method. Therefore, further experiments at the molecular, cellular, and organismal levels are necessary to validate our findings and explore their clinical applications.

## Data Availability

The datasets generated and/or analyzed during the current study are available in the GEO (http://www.ncbi.nlm.nih.gov/geo/).

## Abbreviations

BC: Breast cancer
DEGs: Differentially expressed genes
GEO: Gene Expression Omnibus
DAVID: The Database for Annotation, Visualization and Integrated Discovery
STRING: Search Tool for the Retrieval of Interaction Gene/Proteins
PPI: Protein-protein interaction
IHC: Immunohistochemistry
IARC: International Agency for Research on Cancer
BRCA1: Breast cancer gene 1
BRCA2: Breast cancer gene 2
bcl-2: B-cell lymphoma 2
p53: protein 53
EGFR: Epidermal growth factor receptor
GO: Gene Ontology
KEGG: Kyoto Encyclopedia of Genes and Genomes
MF: Molecular function
CC: Cellular component
BP: Biological process
TCGA: The Cancer Genome Atlas
ICGC: International Cancer Genome Consortium
UALCAN: The University of Alabama at Birmingham Cancer data analysis Portal
GEPIA: Gene Expression Profiling Interactive Analysis
RACGAP1: Rac GTPase-activating protein 1
SPAG5: Sperm-associated antigen 5
KIF20A: Member of the driving protein family 20A
